# Supplementary material for: A Study on the Time–Effect and Dose–Effect Relationships of Polysaccharide from Opuntia dillenii against Cadmium-Induced Liver Injury in Mice
Source: Foods. 2022 May 4;11(9):1340. doi: 10.3390/foods11091340 (PMC9100615; doi:10.3390/foods11091340)
Supplement: Supplementary file 1 [file foods-11-01340-s001.zip › foods-1692580-supplementary.pdf]

foods

Supplementary materials

A Study on Time–Effect and Dose–Effect Relationships of Polysaccharide from *Opuntia Dillenii* against Cadmium-Induced Liver Injury in Mice

Ting Liu [1,2], Bianli Li [1,2], Xin Zhou [1,2*], Huaguo Chen [1,2*]

[1.] *Key Laboratory for Information System of Mountainous Areas and Protection of Ecological Environment, Guizhou Normal University, Guiyang 550001, China*

[2.] *Guizhou Engineering Laboratory for Quality Control & Evaluation Technology of Medicine, Guizhou Normal University, Guiyang 550001, China*

Corresponding author

Guizhou Normal University, 116 Baoshan North Rd., Guiyang, 550001, China

*Tel.: +86-851-8669-0018; Fax: +86-851-8669-0018

E-mail address for Xin Zhou alice9800@sina.com; E-mail address for Huaguo Chen chenhuaguo1981@163.com.

Contents

Table S1. Comparison of mouse hair and eyes between blank group and model group. 3

Table S2: Visual display of liver appearance...4

Table S3: Hair changes in ODPC group mice during 1-5 weeks of ODP administration. 4

Table S4. Liver appearance changes 1-5 weeks after administration of ODP. 5

Table S5. Effect of ODP on body weight in cadmium-induced liver injury, X ± SD (g, n=6) 5

Table S6. Effect of ODP on liver index in cadmium-induced liver injury, X ± SD (%, n=6) 5

Table S7. Effect of ODP on HGB activity in cadmium-induced liver injury, X ± SD (g/L, n=6) 6

Table S8. Effect of ODP on MCH activity in cadmium-induced liver injury, X ± SD (P g, n=6) 6

Table S9. Effect of ODP on MCHC activity in cadmium-induced liver injury, X ± SD (g/L, n=6) 6

Table S10. Effect of ODP on RBC activity in cadmium-induced liver injury, X ± SD ($\times 10^{12}$/L, n=6) 6

Table S11. Effect of ODP on HCT activity in cadmium-induced liver injury, X ± SD (%, n=6) 7

Table S12. Effect of ODP on MCV activity in cadmium-induced liver injury, X ± SD (f L, n=6) 7

Table S13. Effect of ODP on WBC activity in cadmium-induced liver injury, X ± SD ($\times 10^{9}$/L, n=6) 7

Table S14. Multivariate statistical analysis of peripheral blood at 1-5 weeks 7

(n=6 for each week) 7

The table S1-S4 show the visual changes in the appearance of the hair and liver of mice. Tables S5-S13 provide data support for the graph in the manuscript. NC: normal control group, MC: model control group, YC: positive control group, and ODPC: ODP administration group. Data of 6 mice were selected from each group, and the data are expressed as mean ± standard deviation. Similarly, Table S14 shows a multivariate statistical analysis of peripheral blood at 1-5 weeks. The original data of 6 mice were selected for every group for a total of 5 weeks.

Note: *Compared with blank control group, #Compared with model control group; *P<0.05, **P<0.01; #P<0.05, ##P<0.01.

Table S1. Comparison of mouse hair and eyes between blank group and model group.

	Groups		
NC	MC	NC	MC
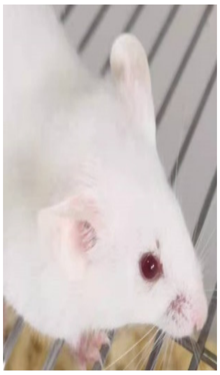	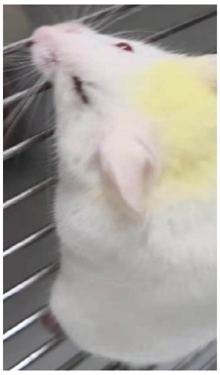	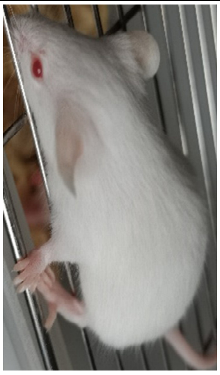	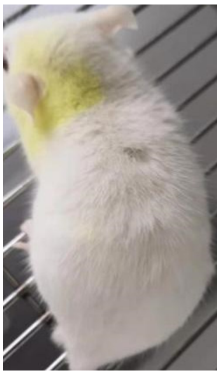

NC: normal control group, MC: $CdCl_2$-treated group.

Table S2: Visual display of liver appearance.

Groups		
NC	MC	YC
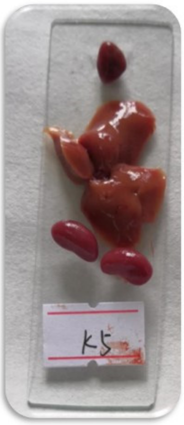	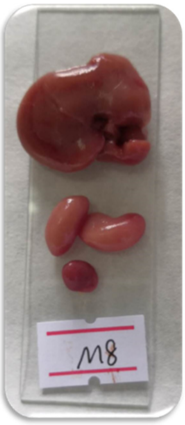	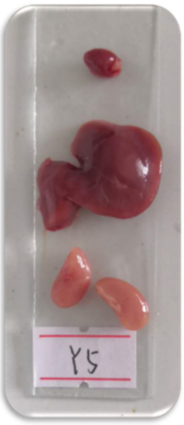

NC: normal control group, MC: $CdCl_2$-treated group, YC: positive-treated group.

Table S3: Hair changes in ODPC group mice during 1-5 weeks of ODP administration.

ODPC group				
Week1	Week2	Week3	Week4	Week5
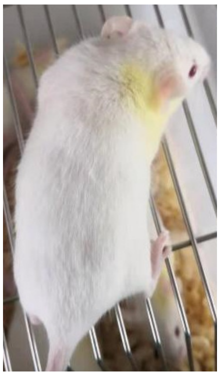	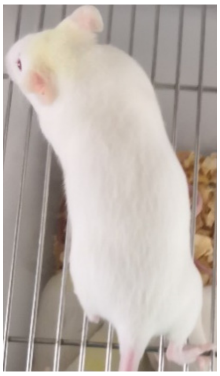	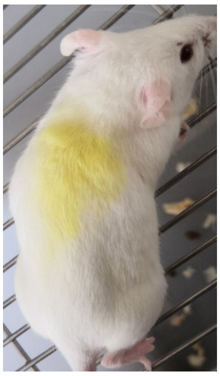	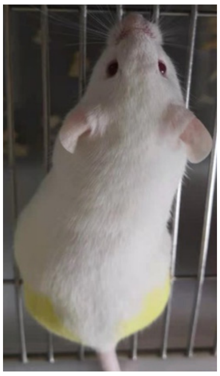	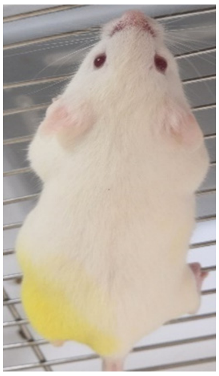

Table S4. Liver appearance changes 1-5 weeks after administration of ODP.

ODPC group				
Week1	Week2	Week3	Week4	Week5

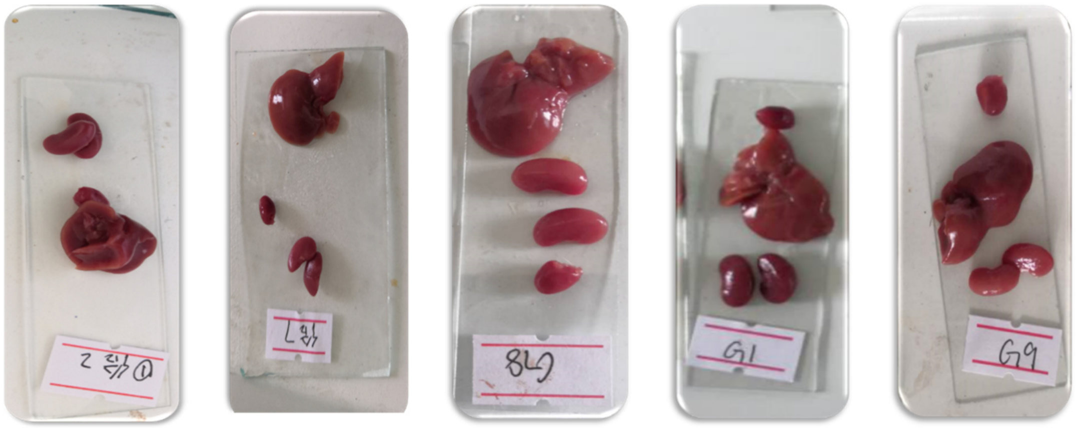

Table S5. Effect of ODP on body weight in cadmium-induced liver injury, $\bar{X}$ ± SD (g, n=6)

Group/Time	Week0	Week1	Week2	Week3	Week4	Week5
NC	22.35±1.73	25.18±0.71	30.00±2.18	35.58±1.59	40.02±1.62	44.90±3.86
MC	22.42±1.22	21.98±1.27**	24.13±1.30**	24.77±1.87**	29.57±4.66**	37.23±1.03**
YC	22.30±0.75	20.90±1.39	27.40±2.02##	28.15±2.00#	33.13±2.92	41.78±2.82#
ODPC	22.15±1.09	22.58±0.36	26.43±1.00#	27.47±1.33#	32.78±3.69	41.13±3.17#

NC: normal control group, MC: $CdCl_2$-treated group, YC: positive-treated group, ODPC: ODP-treated group. * Compared with normal control group (NC group), # compared with model control group (MC group). ** $p < 0.01$; # $p < 0.05$, ## $p < 0.01$.

Table S6. Effect of ODP on liver index in cadmium-induced liver injury, $\bar{X}$ ± SD (%, n=6)

Group/Time	Week1	Week2	Week3	Week4	Week5
NC	3.30±0.38	3.64±0.10	3.90±0.11	3.57±0.19	3.25±0.17
MC	4.70±0.28**	5.13±0.21**	6.63±0.66**	6.80±1.38**	4.71±0.39**
YC	4.25±0.11#	4.48±0.11##	5.34±0.32##	5.56±0.49#	4.46±0.21
ODPC	4.26±0.18	4.61±0.16#	5.77±0.48##	5.80±0.47#	4.46±0.18

NC: normal control group, MC: $CdCl_2$-treated group, YC: positive-treated group, ODPC: ODP-treated group. * Compared with normal control group (NC group), # compared with model control group (MC group). ** $p < 0.01$; # $p < 0.05$, ## $p < 0.01$.

Table S7. Effect of ODP on HGB activity in cadmium-induced liver injury, $\bar{X}$ ± SD (g/L, n=6)

Group/Time	Week1	Week2	Week3	Week4	Week5
NC	139.03±4.35	155.50±4.31	148.23±4.34	150.35±5.74	144.72±4.34
MC	125.77±10.03*	136.33±3.81**	128.57±4.71**	130.37±21.16*	115.47±6.13**
YC	121.48±7.49	143.50±9.96	146.12±7.82##	135.07±8.19	129.03±5.24##
ODPC	134.33±10.65	143.67±6.37	142.37±5.71##	146.88±7.42#	132.93±4.08##

NC: normal control group, MC: $CdCl_2$-treated group, YC: positive-treated group, ODPC: ODP-treated group. * Compared with normal control group (NC group), # compared with model control group (MC group). * $p < 0.05$, ** $p < 0.01$; # $p < 0.05$, ## $p < 0.01$.

Table S8. Effect of ODP on MCH activity in cadmium-induced liver injury, $\bar{X}$ ± SD (P g, n=6)

Group/Time	Week1	Week2	Week3	Week4	Week5
NC	17.82±0.62	16.03±1.80	17.47±0.43	17.05±0.68	17.10±0.19
MC	17.63±0.66	14.20±0.32**	14.77±0.45**	15.20±0.43**	15.42±0.24**
YC	17.37±0.41	14.97±0.22	16.55±0.12##	15.87±0.84	15.52±0.45
ODPC	17.42±0.92	14.96±0.30	16.15±0.49##	15.52±0.37	15.63±0.18

NC: normal control group, MC: $CdCl_2$-treated group, YC: positive-treated group, ODPC: ODP-treated group. * Compared with normal control group (NC group), # compared with model control group (MC group). ** $p < 0.01$; ## $p < 0.01$.

Table S9. Effect of ODP on MCHC activity in cadmium-induced liver injury, $\bar{X}$ ± SD (g/L, n=6)

Group/Time	Week1	Week2	Week3	Week4	Week5
NC	333.33±8.10	339.80±18.40	339.33±9.84	336.67±6.62	336.50±6.13
MC	321.33±10.40	322.00±4.94*	320.17±4.52**	331.67±6.62	323.67±7.99**
YC	329.17±7.03	334.60±3.01	338.83±5.01##	335.33±11.02	324.50±4.03
ODPC	322.17±13.92	328.80±1.60	337.50±6.75##	335.50±10.93	327.17±4.45

NC: normal control group, MC: $CdCl_2$-treated group, YC: positive-treated group, ODPC: ODP-treated group. * Compared with normal control group (NC group), # compared with model control group (MC group). * $p < 0.05$, ** $p < 0.01$; ## $p < 0.01$.

Table S10. Effect of ODP on RBC activity in cadmium-induced liver injury, $\bar{X}$ ± SD ($\times 10^{12}$/L, n=6)

Group/Time	Week1	Week2	Week3	Week4	Week5
NC	8.52±0.20	8.80±0.33	8.60±0.23	8.88±0.18	8.61±0.27
MC	8.11±0.56	7.65±0.39**	8.51±0.30	8.55±1.31	7.90±0.45**
YC	7.97±0.41	8.00±0.57	8.59±0.49	8.88±0.55	8.34±0.39
ODPC	8.68±0.32#	8.03±0.33	8.57±0.36	8.84±0.67	8.56±0.19##

NC: normal control group, MC: $CdCl_2$-treated group, YC: positive-treated group, ODPC: ODP-treated group. * Compared with normal control group (NC group), # compared with model control group (MC group). ** $p < 0.01$; # $p < 0.05$, ## $p < 0.01$.

Table S11. Effect of ODP on HCT activity in cadmium-induced liver injury, $\bar{X}$ ± SD (%, n=6)

Group/Time	Week1	Week2	Week3	Week4	Week5
NC	42.62±1.44	45.18±1.83	44.28±0.72	43.90±1.83	43.35±0.59
MC	39.05±2.35*	38.55±1.62**	39.55±0.88**	39.17±5.11*	35.47±1.67**
YC	36.88±2.60	40.25±3.32	42.37±1.67##	40.55±1.60	39.78±1.76##
ODPC	41.58±2.24	40.98±1.96	41.23±1.87	43.45±2.52#	41.37±0.77##

NC: normal control group, MC: $CdCl_2$-treated group, YC: positive-treated group, ODPC: ODP-treated group. * Compared with normal control group (NC group), # compared with model control group (MC group). * $p < 0.05$, ** $p < 0.01$; # $p < 0.05$, ## $p < 0.01$.

Table S12. Effect of ODP on MCV activity in cadmium-induced liver injury, $\bar{X}$ ± SD (f L, n=6)

Group/Time	Week1	Week2	Week3	Week4	Week5
NC	51.51±2.26	51.37±2.73	50.83±0.58	50.67±1.65	51.83±0.55
MC	52.06±1.45	47.50±0.57**	45.75±1.36**	46±1.80**	47.42±0.97**
YC	49.95±1.23	48.75±0.96	48.52±0.20##	47.23±1.59	47.98±0.93
ODP	48.25±1.88	49.58±0.59#	47.52±0.81#	46.72±0.58	49.08±1.54#

NC: normal control group, MC: $CdCl_2$-treated group, YC: positive-treated group, ODPC: ODP-treated group. * Compared with normal control group (NC group), # compared with model control group (MC group). ** $p < 0.01$; # $p < 0.05$, ## $p < 0.01$.

Table S13. Effect of ODP on WBC activity in cadmium-induced liver injury, $\bar{X}$ ± SD (×10⁹/L, n=6)

Group/Time	Week1	Week2	Week3	Week4	Week5
NC	3.46±0.66	3.93±1.05	3.90±2.50	3.53±0.40	4.47±0.98
MC	6.4±1.46**	4.64±0.60	5.07±1.45	5.43±1.96*	8.37±2.29**
YC	4.05±0.59#	3.33±0.97	4.65±1.15	5.25±1.37	5.10±0.99##
ODPC	4.98±1.25#	4.29±0.80	4.60±1.07	4.60±1.60	5.90±0.89#

NC: normal control group, MC: $CdCl_2$-treated group, YC: positive-treated group, ODPC: ODP-treated group. * Compared with normal control group (NC group), # compared with model control group (MC group). * $p < 0.05$, ** $p < 0.01$; # $p < 0.05$, ## $p < 0.01$.

Table S14. Multivariate statistical analysis of peripheral blood at 1-5 weeks

(n=6 for each week)

Group/Index	HGB	MCH	MCHC	RBC	HCT	MCV	WBC
week1 k1	144.00	17.80	329.00	8.89	45.00	57.20	5.10
week1 k2	144.00	16.90	328.00	8.35	43.90	50.70	4.80
week1 k3	137.60	17.60	331.00	8.34	41.20	53.90	3.70
week1 k4	131.30	18.60	351.00	8.68	41.00	54.80	4.90
week1 k5	139.70	18.60	333.00	8.48	42.70	57.20	3.40
week1 k6	137.60	17.40	328.00	8.37	41.90	53.30	4.90
week2 k1	151.00	15.20	331.00	8.66	43.00	50.10	1.75

week2 k2	151.00	14.60	330.00	8.57	45.80	48.90	3.74
week2 k3	163.00	15.30	328.00	9.13	47.60	50.70	4.17
week2 k4	159.00	15.70	328.00	9.01	47.00	52.20	4.35
week2 k5	155.00	20.00	376.00	9.16	44.90	57.00	4.65
week2 k6	154.00	15.40	337.00	8.26	42.80	49.30	4.94
week3 k1	145.40	17.50	335.00	9.06	43.40	51.50	2.60
week3 k2	146.90	17.60	342.00	8.49	45.10	51.30	2.50
week3 k3	149.80	17.30	327.00	8.52	43.70	51.10	2.50
week3 k4	144.70	16.90	337.00	8.56	45.20	50.90	9.30
week3 k5	157.20	18.30	359.00	8.30	43.70	50.40	2.30
week3 k6	145.40	17.20	336.00	8.68	44.60	49.80	4.20
week4 k1	160.20	18.30	336.00	8.74	46.90	53.70	3.80
week4 k2	145.10	16.20	332.00	8.88	42.90	48.40	2.70
week4 k3	145.50	16.90	351.00	8.73	43.60	51.60	3.50
week4 k4	152.30	16.60	332.00	8.67	41.40	50.00	3.90
week4 k5	154.20	17.50	333.00	9.06	45.60	49.90	3.50
week4 k6	144.80	16.80	336.00	9.17	43.00	50.40	3.80
week5 k1	148.90	17.40	334.00	9.06	44.50	51.90	4.40
week5 k2	139.30	17.10	337.00	8.28	42.60	51.80	5.70
week5 k3	147.90	16.90	330.00	8.78	42.90	52.90	3.60
week5 k4	141.40	17.00	337.00	8.31	43.80	51.90	4.10
week5 k5	140.70	16.90	332.00	8.57	43.30	51.20	5.80
week5 k6	150.10	17.30	349.00	8.65	43.00	51.30	3.20
week1 M1	129.20	18.00	327.00	8.14	39.50	55.40	8.60
week1 M2	134.10	17.60	329.00	8.59	40.70	53.70	9.30
week1 M3	122.30	18.80	326.00	7.50	37.50	57.70	7.20
week1 M4	131.30	17.00	313.00	8.68	41.90	54.60	6.20
week1 M5	132.70	17.60	331.00	8.51	40.00	53.30	8.10
week1 M6	105.00	16.80	302.00	7.23	34.70	55.70	5.00
week2 M1	138.00	14.60	326.00	7.76	38.60	47.80	4.06
week2 M2	138.00	14.00	319.00	7.28	36.40	47.50	4.09
week2 M3	132.00	13.70	318.00	7.79	40.30	46.80	4.82
week2 M4	137.00	14.10	328.00	8.40	38.60	48.00	4.82
week2 M5	132.00	14.20	317.00	7.31	36.70	46.70	5.39
week2 M6	143.00	14.60	328.00	7.37	40.70	48.20	5.67
week3 M1	132.70	15.60	328.00	8.90	40.40	47.60	8.20
week3 M2	130.90	14.50	322.00	8.83	40.70	44.30	4.70
week3 M3	122.60	14.20	321.00	8.24	39.10	44.30	4.30
week3 M4	130.10	14.80	319.00	8.63	38.90	47.50	4.60
week3 M5	121.60	15.00	313.00	8.36	38.20	45.10	4.90
week3 M6	133.50	14.50	318.00	8.11	40.00	45.70	3.70
week4 M1	125.20	15.30	326.00	8.18	38.40	47.00	6.50

week4 M2	117.40	14.50	294.00	8.09	39.80	49.20	3.20
week4 M3	113.20	15.60	337.00	7.22	33.60	46.40	4.80
week4 M4	163.40	15.10	344.00	10.78	47.40	44.00	7.00
week4 M5	108.00	14.90	330.00	7.23	32.70	45.30	2.90
week4 M6	155.00	15.80	359.00	9.79	43.10	44.10	8.20
week5 M1	108.80	15.20	327.00	8.58	33.20	46.70	11.00
week5 M2	111.40	15.50	306.00	7.15	36.30	46.90	7.80
week5 M3	121.60	15.30	327.00	7.93	37.10	48.80	7.30
week5 M4	109.40	15.80	325.00	8.27	33.30	47.80	11.60
week5 M5	124.90	15.60	328.00	7.78	37.50	48.30	4.90
week5 M6	116.70	15.10	329.00	7.71	35.40	46.00	7.60
week1 Y1	133.40	17.50	328.00	8.58	40.60	53.60	5.10
week1 Y2	129.20	17.70	331.00	8.28	39.00	53.70	4.60
week1 Y3	112.90	17.70	340.00	7.36	33.20	52.30	6.20
week1 Y4	118.90	17.70	324.00	7.70	36.60	54.70	5.30
week1 Y5	114.20	16.90	334.00	7.72	34.10	50.80	4.50
week1 Y6	120.30	16.70	318.00	8.20	37.80	52.60	4.60
week2 Y1	132.00	14.80	334.00	7.81	36.30	47.70	2.48
week2 Y2	135.00	15.30	331.00	7.56	37.60	47.80	2.61
week2 Y3	136.00	14.70	334.00	7.18	37.00	49.50	2.74
week2 Y4	148.00	14.80	335.00	8.37	43.30	49.30	2.81
week2 Y5	160.00	15.00	333.00	8.93	44.20	48.00	4.38
week2 Y6	150.00	15.20	340.00	8.14	43.10	50.20	4.98
week3 Y1	153.20	16.70	333.00	8.46	40.90	48.40	3.50
week3 Y2	137.30	16.50	342.00	9.42	44.70	48.60	5.60
week3 Y3	151.80	16.60	344.00	9.10	44.00	48.40	3.90
week3 Y4	156.20	16.40	343.00	8.07	43.10	48.30	6.50
week3 Y5	137.30	16.70	340.00	8.29	40.10	48.90	3.40
week3 Y6	140.90	16.40	331.00	8.21	41.40	48.50	5.00
week4 Y1	134.40	15.10	345.00	8.86	40.70	46.00	5.10
week4 Y2	150.10	15.20	330.00	8.06	43.40	45.40	7.90
week4 Y3	127.40	15.00	317.00	9.82	38.60	45.70	5.80
week4 Y4	140.70	16.40	338.00	8.57	41.60	48.60	3.50
week4 Y5	126.70	16.20	351.00	8.70	39.40	48.20	4.40
week4 Y6	131.10	17.30	331.00	9.27	39.60	49.50	4.80
week5 Y1	125.90	16.00	331.00	7.82	38.00	48.60	6.00
week5 Y2	136.50	16.10	326.00	8.44	41.80	49.60	3.50
week5 Y3	131.60	15.60	325.00	8.41	40.40	48.10	6.20
week5 Y4	124.70	14.90	320.00	8.64	39.30	46.80	5.60
week5 Y5	133.70	15.00	319.00	8.89	41.90	47.20	4.10
week5 Y6	121.80	15.50	326.00	7.85	37.30	47.60	5.20
week1 G1	136.90	17.50	333.00	8.80	41.10	52.80	5.60

week1 G2	124.40	17.20	304.00	8.20	40.80	56.70	4.60
week1 G3	138.30	17.00	310.00	9.09	44.50	55.10	7.00
week1 G4	122.30	16.10	316.00	8.57	38.60	51.10	6.70
week1 G5	154.20	19.20	345.00	9.00	44.60	55.80	7.70
week1 G6	129.90	17.50	325.00	8.40	39.90	54.00	4.30
week2 G1	149.00	15.10	328.00	8.10	42.70	50.00	3.26
week2 G2	141.00	14.90	330.00	7.68	38.70	48.30	3.41
week2 G3	155.00	15.10	329.00	7.77	40.50	49.80	4.16
week2 G4	140.00	15.10	331.00	7.73	44.40	49.60	4.36
week2 G5	136.00	15.30	327.00	8.51	39.40	49.80	5.21
week2 G6	141.00	14.40	327.00	8.39	40.20	50.00	5.36
week3 G1	147.40	16.00	345.00	8.39	38.90	46.40	5.20
week3 G2	142.80	15.80	333.00	8.54	41.80	47.10	6.50
week3 G3	148.10	17.20	340.00	8.45	42.00	49.00	3.40
week3 G4	135.00	16.20	345.00	8.17	44.40	47.10	4.20
week3 G5	134.30	15.90	336.00	8.54	39.10	47.70	3.50
week3 G6	146.60	15.80	326.00	9.34	41.20	47.80	4.80
week4 G1	144.50	15.70	340.00	9.18	42.40	46.40	3.60
week4 G2	146.70	15.00	341.00	8.66	42.90	46.60	2.50
week4 G3	148.90	15.50	354.00	9.70	42.00	47.00	6.60
week4 G4	146.70	15.30	332.00	9.47	44.10	46.60	3.20
week4 G5	134.60	15.40	323.00	7.79	48.60	47.80	5.20
week4 G6	159.90	16.20	323.00	8.26	40.70	45.90	6.50
week5 G1	129.80	15.70	337.00	8.58	41.00	47.90	5.30
week5 G2	126.20	15.60	326.00	8.81	39.90	52.30	7.10
week5 G3	138.00	15.50	326.00	8.77	42.30	48.10	5.10
week5 G4	136.60	15.50	325.00	8.42	42.00	47.90	5.30
week5 G5	135.10	16.00	325.00	8.50	41.60	49.50	5.40
week5 G6	131.90	15.50	324.00	8.26	41.40	48.80	7.20

HGB: hemoglobin, MCH: mean hemoglobin content , MCHC: mean hemoglobin concentration, RBC: red blood cell, HCT: hematocrit, MCV: mean red blood cell volume, WBC: white blood cells
